# Biosensor Strategies for Rapid African Swine Fever Virus Detection

**DOI:** 10.1021/acsomega.5c03168

**Published:** 2025-09-08

**Authors:** Chelsie Boodoo, Evangelyn C. Alocilja

**Affiliations:** Department of Biosystems and Agricultural Engineering, 3078 Michigan State University, East Lansing, Michigan 48824, United States

## Abstract

African swine fever (ASF) is a lethal, highly contagious disease that severely threatens global pig populations and the pork industry. Conventional diagnostics based on virus isolation, ELISA, or quantitative PCR are accurate but require specialized laboratories and hours to days of turnaround, creating a demand for pen-side alternatives. Biosensors address this need, yet ASF virus (ASFV) devices remain at an early stage, with limited field validation and few standardized protocols. This review analyzed forty-one ASFV biosensors reported between 2014 and 2025, grouping them by biological receptor (antibody, enzyme, or nucleic acid) and by transduction mechanism (optical, mass-based, or electrochemical). Reported limits of detection extend from five copies per microliter for a CRISPR–Cas12a fluorescence assay^1^ to 0.5 ng mL^–1^ for a piezoelectric cantilever antigen sensor,^2^ with assay times ranging from 5 to 45 min. This review examined the sensitivity, specificity, and assay times, highlighting persistent deficits in cross-reactivity analyses and environmental stress testing. Result of the analysis underscores an urgent need for harmonized testing conditions and robust comparative studies to bridge the gap between proof-of-concept demonstrations and commercially viable products. By contrast of current ASFV biosensor research with more advanced platforms targeting other viral pathogens, practical design strategies and future directions to enhance accuracy, scalability, and cost-effectiveness were identified. Ultimately, this Perspective provides a roadmap for advancing ASFV biosensor technology to mitigate outbreaks more effectively and safeguard global pork production.

## Graphical abstract

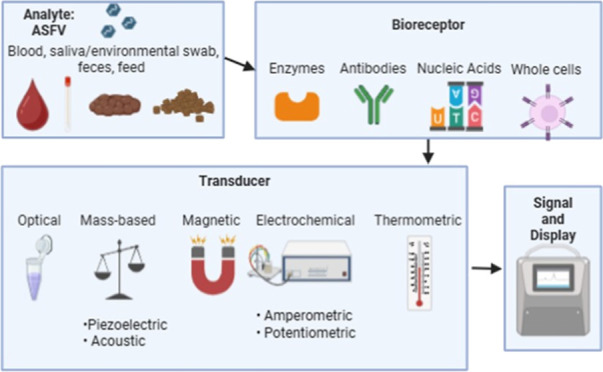

## Introduction

African swine fever (ASF) is a viral hemorrhagic disease with high lethality in both feral and domestic swine.^3^ First identified in Kenya in 1909, ASFV remains endemic in sub-Saharan Africa and has spread across Europe, Asia, and the Caribbean, causing devastating economic losses in the pork industry.^4^ The disease’s impact on global trade is extensive. For instance, a 2017 outbreak in China resulted in over 20 million USD in losses within two months. If ASFV were introduced into the United States, it could jeopardize the pork industry worth approximately 15 billion USD.^5^ ASFV’s incubation period ranges from 4 to 19 days.^6^ Transmission occurs through contaminated feed, fomites, pork products, and blood-feeding invertebrates, making early detection critical for containment.^7^ Containment hinges on rapid on-farm diagnostics: the earlier an infected herd is identified, the narrower the culling radius and the lower the market shock.

Conventional laboratory methods such as virus isolation, World Organisation for Animal Health (OIE)-prescribed qPCR, and ELISA offer high sensitivity but require specialized equipment, cold-chain storage, and multihour processing.^8^ These limitations have driven interest in biosensors for ASFV detection. Biosensor platforms that are being explored span biological, optical, mass-based, and electrochemical modalities, which are highlighted in the tables of this paper.

ASFV has a complex structure composed of several proteins, many of which remain poorly characterized in terms of function and mechanism. This knowledge gap has hindered the development of effective vaccines and a limited understanding of ASFV pathogenesis. Investigating the structure and function of these proteins is essential to elucidate the virus’s infectious mechanisms. Nevertheless, significant progress has been made in recent years in defining the roles of key ASFV proteins.

Although ASFV’s double-stranded DNA genome spans approximately 170–190 kilobases, it is not the genome size but rather the high degree of genetic variability, particularly multigene families such as MGF 100, 110, 300, 360, and 505/530, that complicates probe and primer designs. These multigene families vary across genotypes, leaving only a few regions, such as the B646L (p72) and CP204L (p30) genes, that are sufficiently conserved for universal primer or probe design.^9–11^ Consequently, biosensor platforms often target these conserved genes or their associated proteins. For example, Wu et al.^12^ used recombinase-aided amplification combined with a CRISPR–Cas12a lateral flow assay to detect p72 in whole blood at 5 copies/μL within 25 min. Zhao et al.^13^ developed a nanoplasmonic biosensor that quantifies serum p30-IgG with a limit of detection of 0.78 ng/mL in under 15 min.

Biosensors convert molecular recognition events into quantifiable signals and have demonstrated strong potential for on-site ASFV detection.^14^ Recent advances in nanoplasmonic biosensing and isothermal amplification assays have shown strong potential for point-of-care ASFV screening, significantly reducing detection time to about 20 min with minimal sample preparation.^13^ Recombinase-based isothermal amplification assays offer sensitivity comparable to qPCR and do not require thermal cycling or nucleic acid extraction, features that make it suitable for field deployment.^15,16^

Despite these promising attributes, ASFV-focused biosensors lack the more extensively validated platforms available for viruses such as influenza or SARS-CoV-2, with significant gaps in standardization, large-scale validation, and real-world testing. By contrast, commercial biosensors for some other viral pathogens have already demonstrated robust performance and field readiness, highlighting the relative infancy of ASFV biosensor research. Moreover, many ASFV prototypes have been trialed only in controlled laboratory settings, leaving field performance largely uncharacterized.

This review addresses those gaps by analyzing the current landscape of ASFV biosensors, highlighting both their strengths and their limitations. By critically comparing sensor modalities (optical, mass-based, and electrochemical), this paper aims to provide a strategic roadmap for optimizing ASFV detection in both laboratory and resource-constrained settings, ultimately advancing global disease control efforts against ASF.

## Biological Receptor Biosensors

Biosensors contain biorecognition elements known as biological receptors, which can be antibody-, enzyme-, DNA-, or CRISPR-based.^17^ Biological-receptor biosensors translate a molecular recognition event, antibody–antigen binding, enzyme-mediated amplification, nucleic acid hybridization, or CRISPR collateral cleavage into an electrical or optical signal.

### Antibody-Based Formats

Lateral-flow immunoassays (LFIAs) targeting structural ASFV proteins remain the most mature antibody-based biosensor platforms. These assays typically rely on polyclonal or monoclonal antibodies directed at viral markers, such as p72, p30, and VP73, and have shown strong field applicability. One strip targeting p72 reported a detection limit of 15 copies per reaction in blood and tissue matrices, while another targeting p30 detected 2.16 ng of antigen within 10 min postextraction.^16^ A separate GNP-based test strip targeting p30 detected 2.16 ng of p30 in plasma and tissue samples within 10 min post-DNA extraction.^18^

Some platforms integrate smartphone compatibility. For example, a p30-IgG LFIA achieved 0.78 ng/mL sensitivity in serum and closely matched qPCR results across 163 field samples.^12^ Smartphone-read LFIAs have also been reported, such as a p30-IgG strip achieving 0.78 ng/mL sensitivity in serum with strong agreement to qPCR across 163 samples.^13^

Table 1 includes LFIAs that utilize p30,^13,18,19^ p72,^20–22^ and VP73 ^12,23^ as target antigens. Some strips tested matrices beyond serum and blood, such as kitchen waste and pork products.^21^ While polyclonal antibodies remain cost-effective, they face issues of variability and lower specificity. Recombinant formats improve performance but require advanced protein expression systems and purification infrastructure.^24^

**Table 1:** Overview of Biological and Optical ASFV Biosensor**s** ^a^

technique	sensitivity	output	matrix	total assay time	nontarget	target gene	ref
GNP-based, lateral flow strip	15 copies/reaction	colorimetric	plasmid, tissue samples (liver, spleen, kidney, lung, pancreas, intestine, lymph and muscle) and blood	1 h: PCR, then 5 min for the strip	CSFV, PRRSV, PRV, PCV-2, FMDV, PPV, and PEDV	p72	^20^
GNP-based, test strip	2.16 ng of p30	colorimetric	sera, plasma, anticoagulant-treated blood, and tissue	10 min after DNA extraction	PRV Bartha-K61, CSFV, PRRSV, SVA, PCV-2, PEDV, TGEV, PPV	p30	^18^
Cas12, fluorescence immunoassay, GNP-based, lateral flow strip, test strip	2.5 × 10^–15^ M	fluorescence and colorimetric	swine blood samples	2 h	Escherichia coli, Pseudomonas aeruginosa, Staphylococcus aureus	VP73	^12^
fluorescence immunoassay	0.015 ng/mL (dynamic range 0.24–500 ng/mL)	fluorescence	nose and mouth discharge	45 min	TGEV, PRCV, PHEV, PCV	p30	^19^
GNP-based, lateral flow strip	1 × 102 copies/reaction	colorimetric	kitchen waste, swill samples, environment samples from meat stalls at farmer’s markets and pork product samples	15 min	CSFV/Thiverval, RVA/NX, PEDV/CV777, TGEV/HUA, PRRSV/SCcd17 and PRV/Bartha	K205R gene of ASFV-SY1	^21^
GNP-based, lateral flow strip	1.75 × 102 copies/μL	colorimetric	blood	30 min	CSFV	p72	^42^
GNP-based, lateral flow strip	10 copies/μL	colorimetric	blood	10–15 min	CSFV, PRRSV, PEDV, PRV, PCV2	B646L	^22^
assay-linked immunosorbent assay (AlphaLISA), fluorescence Immunoassay	≥0.78 ng/mL	colorimetric	serum	2 h	CSFV, PRV, PRRSV, PPV, PCV2, PEDV	p72	^34^
Cas12, fluorescence immunoassay	2 copies/μL reaction	fluorescence	plasmid	1–2 h	PRRSV, H1N1-PR8, J.E.V., and PDCoV	p72	^1^
isothermal amplification	10 copies	fluorescence	oral swabs and blood samples	20 min	PCV2, PPV, PRV, CSFV, PRRSV	B646L and EP402R gene	^43^
isothermal amplification	15.21 copies μL^–1^	colorimetric and turbidity	blood or tissue	32 min	CSFV, PCV, P.R.V., PPRV	p72	^27^
Cas12	50 pM	colorimetric/fluorescence	clinical tissue samples, including the spleen and kidney	30 min	P.R.V., HBV	N/A	^32^
GNP-based, lateral flow strip	1:256	colorimetric	serum	5–10 min	PRRSV, FMDV-A, FMDV-O, PCV-2, CSFV	p30 and p72	^23^
fluorescence immunoassay, chemiluminescent immunoassay (CLIA)	1:128	fluorescence	serum	>2 h	PRV, PPV, PRRSV, JE, CSFV	p54	^36^
fluorescence immunoassay, SYBR Green I-staining R.P.A. (RPAS), recombinase polymerase amplification (RPA)	103 copies/μL	fluorescence	plasmid, spleen and kidney specimens, sera, nasal swabs, anal swabs, and feces	15 min	PRV, JEV, PEDV, PRRSV, PPV	p72	^28^
lateral flow strip	104 HAU	colorimetric	blood	10 min	none	p72	^44^
GNP-based, lateral flow strip	1:409,600	colorimetric	serum	10 min	PRV, CSFV, PCV2, PRRSV, PPV, FMDV	p72	^24^
isothermal amplification	10 copies/reaction	colorimetric, fluorescent	plasmid	45 min	CSFV, HP-PRRSV, PCV2, PCV3, JEV, PPV, PEDV, PRV	p72	^25^
fluorescence immunoassay, isothermal amplification	30–50 copies/μL	fluorescent	synthetic ASFV DNA and mock DNA	70 min	PRV, PCV2, PPV	B646L, B962L, C717R, and D1133L	^45^
fluorescence immunoassay, GNP-based	1:16000	colorimetric	serum	20 min	PRRSV, CSFV, PCV-2, PEDV, TGEV, JEV, H1N1, and PRV	p30	^13^
magnetofluidics	5 copies/μL	ct values	serum, environmental swabs, homogenized tissue, swine feed	30 min	CSFV, PRRSV, PRV, PPV, PCV2	N/A	^46^
lateral flow strip, RPA	150 copies per reaction	fluorescent	spleen and blood	10 min	PRRSV/CH1R, CSFV/Shimen, PRV/JL14, JEV/SA14–14–2, PEDV/CV777, and PCV2b-SD12	p72	^26^
fluorescence immunoassay, lateral flow strip	1:64000	fluorescent	serum	25 min	PCV2, FMDV, CSFV, HPS2, HPS8, HPS10, Mhp, Mhs, Mf, PRRSV, PRV, H3N2	p30 and p54	^35^
fluorescence immunoassay, isothermal amplification	10 copies/μL	fluorescent	whole blood and muscle tissue	30 min	PRPSV, PRV, TGEV, H1N1, PTV, SSP	N/A	^47^
chemiluminescence	1:256	luminescence	serum	3 h	SVA, CSFV, PRRSV, TGEV, PEDV, PRV, PCV2, FMDV type O and A	p30	^29^
chimeric DNA/LNA, SPR	178 copies/μL	arcsec	blood	5 min	PCV, CSFV	vp72	^30^
DNA and magnetic nanomaterials	26.1 fm	colorimetric	blood and serum	30 min	Two DNA targets with random sequences	p72	^48^
lateral flow strip, LAMP and CRISPR	10 copies/μL	colorimetric	blood	45 min	CSFV, PEDV, PRV, PPV, PRRSV, PCV	N/A	^33^
LAMP on a disposable screen-printed carbon electrode	3.0 × 10^–13^ g/L (0.48 copies/μL)	colorimetric	serum	42 min	ASFV p12, p14, and p17	p54	^31^

a This table combines the biological and optical ASFV biosensors because many of them use both methods. The techniques, sensitivity, output, matrix, total assay time, and nontargets used for determining specificity and target genes are shown in the reference papers. Detection units differ by assay type and are reported as listed in the original publications.

## Enzyme-Driven Isothermal Amplification

Enzyme-based biosensors require careful immobilization to preserve the enzyme functionality and bioactivity. These biosensors commonly incorporate optical transducers such as surface plasmon resonance (SPR), luminescence, or chemiluminescence. In principle, enzyme-based biosensors offer distinct advantages such as low cost, high specificity, and strong selectivity.

ASFV diagnostics have rapidly embraced isothermal methods because enzymes confer attomole-level sensitivity at a single temperature. For instance, a LAMP-based test using neutral red dye achieved 10 copies/μL detection in plasmid samples within 35 min,^25^ while RPA combined with lateral flow formats achieved 150 copies per reaction in tissue samples.^26^

Recent innovations aim to miniaturize and adapt these techniques to field conditions. Miao et al.^26^ used a lateral flow LAMP assay to detect ASFV in serum samples at 10 copies/μL in 35 min, while Cao et al.^27^ incorporated carbon nanodots with LAMP for fluorescence-based detection. Other adaptations, such as Zhang’s^28^ SYBR Green I-based LAMP, enabled visual detection in under an hour. Beyond amplification, enzyme-linked assays such as chemiluminescent immunoassays have also been developed. For example, Shi et al. demonstrated antibody detection in serum using magnetic particles and chemiluminescence, supporting rapid and highly sensitive readouts.^29^

### DNA-Hybridization Sensors

Hybridization-based biosensors leverage the principle of complementary base pairing to detect ASFV nucleic acids without bulk amplification. Biagetti et al.^30^ immobilized chimeric DNA/LNA probes on a SPR biosensor and achieved a detection limit of 178 copies/μL for ASFV VP72 in serum. The label-free design enabled real-time monitoring and required no enzymatic amplification, offering the potential for simplified diagnostics. Yuan et al. took a different approach using thiolated DNA probes on disposable electrochemical chips to detect p54 at 10 fM in serum.^31^ Though not purely hybridization-based, the specificity of probe–target binding places this design within the same diagnostic framework.

These hybridization sensors^30,31^ which are highlighted in Table 1, operate at room temperature and bypass the cold chain and enzymatic requirements. Still, most systems have not been comprehensively tested against viruses such as PRRSV or CSFV, limiting claims of diagnostic specificity in field conditions.

### CRISPR/Cas-Hybrid Systems

Several ASFV biosensors now incorporate CRISPR/Cas12a for improved specificity and a rapid readout. Ki et al.^32^ designed a colorimetric system using Cas12a to detect ASFV p72 visually, without complex instruments. In further advancement, Chen et al. developed an instrument-free format that detects ASFV directly in raw blood within 30 min^33^

These CRISPR-based biosensors are shown in Table 1 under targets such as p72,^32,33^ spanning both fluorescence and lateral flow formats. Although the sensitivity is impressive, widespread adoption is limited by the potential for environmental contamination, reagent degradation, and inconsistent performance across different sample types. Future development will depend on achieving robust standardization and validation for use in diverse real-world settings.

## Optical Biosensors

Optical biosensors register analyte binding as a change in fluorescence, chemiluminescence, or plasmonic color or SPR.

### Fluorescence and Chemiluminescence

Fluorescence and chemiluminescence biosensors offer the sensitive detection of ASFV through light-emitting reactions or fluorescent signal changes upon target binding. Tao et al.^1^ used a CRISPR-Cas12a fluorescence assay with nucleic acid amplification to detect p72 in whole blood at 5 copies/μL in just 25 min. Similarly, Chen et al.^34^ developed an AlphaLISA immunoassay for p30 that achieved a 1:64,000 serum dilution sensitivity in 30 min.

Other formats like quantum dot immunosensors (QAIS)^35^ and chemiluminescence assays targeting p30 ^36^ provided comparable results with varying detection times.^35,36^ Zhang et al. utilized SYBR Green I in a LAMP-based assay to detect ASFV DNA in whole blood at 15.21 copies/μL within 32 min, offering a visual, fluorescence-driven alternative suitable for lower-resource settings.^28^

### Plasmonic and Surface-Plasmon-Resonance Sensors

Plasmonic biosensors exploit the shift in light absorption caused by analyte binding on nanostructured surfaces, while SPR monitors changes in the refractive index at a metal–dielectric interface, enabling label-free and real-time detection. Zhao et al. functionalized gold nanoparticles with p30 for serum antibody detection, achieving 0.78 ng/mL sensitivity in 15 min with strong concordance to qPCR.^13^

Multiplex formats have also emerged. Gómez-Gómez et al. developed a photonic integrated circuit (PIC) capable of detecting ASFV alongside five other swine viruses using culture supernatants.^37^ In another application, Yuan et al. used SPR to quantify p72 at 0.1 fM in clinical samples in under 50 min^38^ Finally, Papadopoulou et al. used SPR with DNA/LNA probes to detect VP72 amplicons at 178 copies/μL in serum.^30^

Together, these SPR and plasmonic sensors^13,30,37,38^ underscore optical sensing’s potential for rapid, sensitive, and even multiplex diagnostics. However, many remain confined to controlled laboratory settings due to their optical complexity, surface instability, and susceptibility to temperature or matrix interference. To make these systems viable for farm use, future development must address the issues of sensor durability, affordability, and field calibration. Successes from human point-of-care diagnostics suggest that, with robust packaging and reagent stability, optical biosensors could eventually meet the speed and usability needs of frontline ASFV surveillance.^39–41^

### Mass-Based Biosensors

Mass-based biosensors detect biomolecular interactions by measuring changes in mass on a sensor surface, often using quartz crystal microbalance (QCM), piezoelectric sensors, or magnetoelastic resonance.^50^ These platforms offer real-time, label-free detection and are valuable for monitoring antigen–antibody binding or molecular adsorption without requiring optical or enzymatic tags.^49^

### Quartz Crystal Microbalance and Piezoelectric Sensors

Several early ASFV biosensors relied on QCM technology. Abad et al.^50^ developed a QCM assay to detect antibodies against the ASFV p12 attachment protein in swine serum. Their system demonstrated a reproducible signal response to varying antibody titers. Similarly, Uttenthaler et al.^51^ designed a QCM biosensor that identified ASFV antigen binding through shifts in resonance frequency, confirming mass-based detection capability in whole serum. A related piezoelectric immunosensor by the same group^2^ used immobilized ASFV proteins to detect binding events with high specificity and demonstrated feasibility for point-of-care adaptation.

These mass-sensitive devices typically offer fast response times and can be miniaturized. However, their surface chemistry must be optimized for the consistent immobilization of ASFV antigens or antibodies. Sensitivity may also decline in viscous or particulate-rich samples, which affects frequency shifts unrelated to specific binding.

### Magnetoelastic Resonance Sensors

Recent work by Liu et al.^52^ introduced a flexible magnetoelastic biosensor composed of PDMS, FeSiB alloy, and quantum dots for ASFV detection. This composite film responded to ASFV antigen binding through mechanical resonance changes, which were transduced into electrical signals. The device detected the p72 protein in under 40 min and showed potential for low-cost, field-deployable use.

### Photonic and Integrated Mass Platforms

Manessis et al.^53^ reported a mass-sensitive PIC platform that combined microfluidics and optical interferometry to detect ASFV and classical swine fever virus (CSFV) simultaneously. While primarily optical, the PIC chip’s design also captures biomolecular mass loading as part of its interferometric signal. This system was tested in serum and environmental samples and showed high diagnostic concordance.

These examples from Table 2 illustrate the diversity of ASFV mass-based biosensing approaches, which range from classic piezoelectric crystals^2,50,51^ to modern magnetoelastic films^52^ and photonic circuits.^53^ Although promising, mass-based platforms require careful surface engineering and may be more sensitive to mechanical or environmental disturbances than optical or electrochemical systems.

**Table 2:** Overview of Mass-Based ASFV Biosensor**s** ^a^

technique	sensitivity	output	matrix	total assay time	nontarget	target gene	ref
quartz, immunosensor	0.31 μg/mL serum; 0.1 μg/mL antibody detection	resonance frequency shift	serum	4 days	none	VP73/p72	^2^
quartz, immunosensor, piezoelectric	1:1280	resonance frequency shift	serum	10 min	negative control pig serum	VP73	^51^
quartz, immunosensor, piezoelectric	1/1500	resonance frequency shift	serum	30 min	negative control pig serum	p12	^50^
magnetoelastic	0.83 pg/mL	resonance frequency shift	ASFV antigen	42 min	PCV, PPV, CSFV	p72	^54^
magnetoelastic	0.079 ng/mL	resonance frequency shift and luminescence	oral fluid and serum samples	60 min	PCV, PPV, CSFV	p72	^53^

a The techniques, sensitivity, output, matrix, total assay time, and nontargets used for determining specificity and target genes are shown with the reference papers. Detection units differ by assay type and are reported as listed in the original publications.

## Electrochemical Biosensors

Electrochemical biosensors offer a promising approach for ASFV detection due to their low cost, high sensitivity, and compatibility with portable formats. These sensors convert biochemical interactions into electrical signals using voltammetric, amperometric, impedimetric, or potentiometric readouts. Their flexibility allows for adaptation across different targets, from viral DNA to specific proteins and antibodies.^55^

### Voltammetric and Amperometric Sensors

Voltametric and amperometric formats are among the earliest explored for ASFV. Stiene and Bilitewski^56^ developed a competitive immunoassay using flow injection analysis to detect ASFV antibodies in pig serum. This approach demonstrated accuracy comparable to that of ELISA and helped set the foundation for subsequent electrochemical designs. More recent work by Yuan et al.^31^ introduced a disposable chip integrating rolling circle amplification and gold nanoparticles to detect the p54 gene, achieving sensitive detection at 10 pg/mL. Similarly, Li et al.^35^ created an electrochemical immunosensor using quantum dot microbeads to capture ASFV antibodies in serum with a detection limit of 1.25 ng/mL, with results available in under 30 min.

### Impedimetric Sensors

Impedimetric sensors have also been used for protein detection. Zhang et al.^18^ developed an impedimetric immunosensor targeting the p30 protein, using monoclonal antibodies immobilized on the sensor surface. The system demonstrated high specificity in serum, with an LOD of 2.7 ng/mL, suggesting its potential utility in field-based surveillance. These electrochemical formats, ranging from flow-based immunoassays to impedance and potentiometric sensors, highlight the broad utility of electrical signal transduction for ASFV detection. Table 3 includes these examples, with voltammetric and amperometric approaches,^31,56^ impedimetric sensors,^18^ and emerging potentiometric devices,^52^ each offering unique advantages depending on the target molecule and desired operational setting.

**Table 3:** Overview of Electrochemical ASFV Biosensor**s** ^a^

technique	sensitivity	output	matrix	total assay time	nontarget	target gene	ref
isothermal amplification, electrochemical	0.48 copies/μL	cyclic voltammetry signal	feces	42 min	ASFV p12, p14, and p17	p54	^31^
electrochemical	0.079 ng/mL	resonance frequency shift and luminescence	oral fluid and serum samples	60 min	PCV, PPV, CSFV	p72	^53^
electrochemical	1 ng/mL-1	horseradish peroxidase activity	serum	15 min	none	VP73	^56^
electrochemical and isothermal amplification	0.048 copies/μL	photocurrent intensity	liver tissue	5 min	ASFV p12, p14, and p17	p54	^38^
quartz, immunosensor	0.31 μg/mL serum; 0.1 μg/mL antibody detection	resonant frequency	serum	4 days	none	VP73/p72	^2^
photonic integrated circuit with antibodies	1/20	resonant frequency	cell culture supernatant	30 min	PCV2, PPV, PRRSV, CSFV, SIV	p30	^37^

a The techniques, sensitivity, output, matrix, total assay time, and nontargets used for determining specificity and target genes are shown with the reference papers. Detection units differ by assay type and are reported as listed in the original publications.

## Discussion

Across the 41 ASFV biosensor studies reviewed, reported limits of detection range from 5 copies/μL for a CRISPR–Cas12a fluorescence assay^1^ to 0.5 ng/mL for a piezoelectric cantilever targeting p72^2^. Most platforms deliver results within 5–45 min. Ten studies evaluated ≥50 clinical blood or serum samples, and four reported agreement with the OIE-prescribed qPCR at or above Cohen’s kappa of 0.90.^1,13,17,53^ These figures suggest that many laboratory prototypes already reach the analytical sensitivity of commercial influenza or SARS-CoV-2 tests, although they still lag behind in validation scale and reagent durability.

Only two devices reported stability testing beyond 30 days across a 4–37 °C range,^31,57^ and none provided diagnostic sensitivity/specificity data from the 200+ field samples typically required for regulatory review. Instrument needs remain a barrier: fluorimeters and impedance analyzers add cost and power burden, while instrument-free colorimetric strips can suffer from GNP aggregation after 3 months at ambient temperature.^45^ Although LAMP and CRISPR systems remain highly sensitive, they remain prone to aerosol contamination. This risk can be reduced by using sealed cartridge formats, similar to those employed in commercial COVID-19 diagnostics.^58^

Several technical advances show promise in addressing these gaps. Lyophilized reagent beads, used in SARS-CoV-2 RPA kits,^26^ have extended GNP shelf life to 12 months in preliminary ASFV work.^59^ Wireless magnetoelastic ribbons combining label-free mass sensing with smartphone readout achieved 0.79 ng/mL detection in 25 min and survived 50 bending cycles.^54^ Propidium monoazide (PMA) pretreatment, which suppresses signal from noninfectious particles, can be added to CRISPR workflows for distinguishing viable viruses, improving disinfection efficacy assessments.

Comparison between platforms is complicated due to varied units (copies/μL, ng/mL, HAD_50_/mL) and matrices (serum, blood, tissue, saliva). Future manuscripts should report LODs in copies/μL for DNA or ng/mL for protein and always indicate the matrix tested. Specificity panels should include viruses with overlapping symptoms (e.g., CSFV, PRRSV).^20,44^ Ring trials using ≥200 field samples and parallel qPCR validation are essential for regulatory consideration. At least 6 months of reagent stability under farm-level conditions should also be documented.^60^

Field-ready ASFV biosensors can help shift outbreak control from reactive to proactive. Paired with cloud-linked readers, these devices enable pen-level surveillance and targeted containment, preserving a $15 billion pork industry.^5^ To reach this goal, developers must combine lab sensitivity with ruggedization, validated specificity, and clear reporting. ASFV biosensors could then serve as key components of early warning systems for both domestic and wild pig populations.^61^ The reviewed biosensors demonstrate high sensitivity, but most have not been validated in real-world settings. Sensitivity values vary depending on the platform and sample matrix, making comparison difficult without standardized benchmarks. In contrast, biosensor platforms for influenza and SARS-CoV-2 ^51,62,63^ already benefit from commercial kits and harmonized validation protocols.

Field deployment of ASFV biosensors faces multiple challenges. Many require bulky instruments,^23,27,31,35,44,47^ and fluorescent systems may rely on dyes like SYBR Green I, which can bind nonspecifically to any double-stranded DNA.^22^ To improve assay sensitivity, preamplification steps are often used,^20^ but postamplification contamination remains a concern.^13,24,42^ Reagent stability is also limited. GNPs tend to aggregate after 3 months,^45^ while some SARS-CoV-2 test kits use protective additives to extend shelf life.^60^ Applying these strategies to ASFV biosensors could enhance their viability in resource-limited or rural settings.

Despite these issues, ASFV biosensors remain essential for biosecurity and disease containment. Tools like rope-in-a-bait sampling^64^ enable aggregate surveillance while minimizing contact exposure. Prompt diagnosis helps limit trade disruption and prevents overculling.^65^

Future biosensor development could benefit from pairing with viral characterization tools such as hemadsorption inhibition assays (HADIA), which help distinguish strain serotypes and inform vaccine development.^66^ ASFV’s genomic complexity, including immune-modulatory genes like EP402R and CD2v, poses challenges for biosensor design and robustness.^64,67^ Incorporating detergents for sample inactivation may improve safety and handling without sacrificing sensitivity.^68^ Drawing from Ebola and Nipah virus protocols,^69,70^ inactivation guidelines can streamline ASFV testing under biosafety constraints. PMA pretreatment holds potential for differentiating infectious versus inactivated virus particles,^71^ improving disinfection validation and reducing unnecessary responses. This technique, now common in diagnostics for other high-risk zoonotic pathogens,^46,72,73^ could improve ASFV biosensor accuracy under field conditions. To ensure cross-platform consistency, the sensitivity should be reported using standardized metrics for defined matrices. External reference panels, similar to those used in human diagnostics, allow for performance benchmarking.

ASFV biosensors offer a path to fast, decentralized outbreak detection, preserving trade and animal welfare. Continued progress depends on integrating data systems, standardizing validation protocols, and tailoring devices to their intended use. With these steps, ASFV biosensors can transition from research prototypes to frontline tools for safeguarding swine health and global food security.

## Conclusion

ASFV remains a serious threat to global food security due to the limitations of traditional laboratory diagnostics, which are often expensive and slow and require centralized facilities. The biosensors surveyed in this review demonstrate that ASFV can be detected in as little as five to forty-five minutes, with limits of detection ranging from five copies/μL for nucleic acid targets to 0.5 ng/mL for protein targets.^1,2,27,28^ These results show promise, but variability in sensitivity units and limited field validation still hinder regulatory advancement.

For biosensors to be implemented routinely on farms, future platforms must report sensitivity in consistent units such as copies/μL or nanograms per milliliter and specify the matrix used, whether serum, blood, or environmental samples. Several reviewed studies include preliminary matrix validation and specificity testing against coendemic swine viruses like PRRSV and CSFV,^13,20,24,28^ but few exceed 100 field samples or report shelf life data beyond 30 days. Regulatory clearance will require at least 200 diverse clinical samples, agreement with qPCR, and demonstration of long-term stability under ambient conditions.^23,57^

Some recent advances address these gaps. Lyophilized reagents, as used in commercial SARS-CoV-2 assays,^74^ offer improved reagent stability and eliminate cold-chain needs. Wireless magnetoelastic sensors,^54^ label-free DNA hybridization formats,^75^ and cartridge-integrated CRISPR–Cas12a assays^50^ show potential for reliable deployment in field conditions.

ASFV’s complex genome, including immune-modulatory proteins such as EP402R, complicates biosensor design due to genetic variability and inconsistent epitope exposure.^64,67^ However, several reviewed biosensors successfully targeted conserved genes like B646L (p72) or structural proteins like p30 and VP73, improving cross-genotype detection.^13,20,22,33,59^ By integrating biosensor outputs with cloud-connected readers, applying lyophilized chemistry, and utilizing closed cartridges similar to those in commercial SARS-CoV-2 platforms,^60^ developers can advance ASFV biosensors from bench to barn. In doing so, these technologies could provide veterinarians and farmers with timely, field-deployable diagnostics, helping to limit culling to infected pens, contain outbreaks early, and preserve international pork trade valued at over $15 billion USD.^5^

By integrating biosensor outputs with cloud-connected readers, applying lyophilized chemistry, and utilizing closed cartridges similar to those in commercial SARS-CoV-2 platforms,^76^ developers can advance ASFV biosensors from bench to barn. In doing so, these technologies could provide veterinarians and farmers with timely, field-deployable diagnostics, helping to limit culling to infected pens, contain outbreaks early, and preserve international pork trade valued at over $15 billion USD.^5^

Collectively, the biosensor platforms highlighted here offer robust solutions for early detection, real-time monitoring, and improved control of ASFV, ultimately helping prevent outbreaks and reducing the economic burden on the pig industry. As the next steps, integrating digital technologies, refining reagent stability, and streamlining on-site workflows will be crucial to unlocking the full potential of ASFV biosensors. By learning from well-established platforms for influenza, SARS-CoV-2, and other pathogens,^77–80^ ASFV biosensors can evolve into indispensable tools for safeguarding global swine health and food security.

## Vocabulary

1. African Swine Fever Virus (ASFV): A highly contagious, double-stranded DNA virus that causes African swine fever, a lethal disease in domestic and wild pigs with major economic and food security implications.
2. Biosensor: An analytical device that integrates a biological receptor (e.g., antibody, enzyme, or nucleic acid) with a transducer to detect target analytes rapidly and accurately.
3. Transducer: The component of a biosensor that converts the biological interaction (e.g., binding of a virus to an antibody) into a measurable signal, such as an electrical current or optical readout.
4. CRISPR: An acronym for Clustered Regularly Interspaced Short Palindromic Repeats, a gene-editing and diagnostic technology that uses CRISPR-associated (Cas) proteins to detect or modify specific nucleic acid sequences.
5. Loop-Mediated Isothermal Amplification (LAMP): A DNA amplification method performed at a constant temperature, eliminating the need for thermal cycling and enabling rapid, simple pathogen detection in field settings.
6. Recombinase Polymerase Amplification (RPA): An isothermal nucleic acid amplification technique that uses recombinase to detect DNA or RNA with minimal equipment requirements, making it suitable for point-of-care diagnostics.
7. Surface Plasmon Resonance (SPR): An optical phenomenon employed in biosensors where changes in the refractive index at a metal surface (often gold) are measured to detect binding events between biomolecules.
